# Exploring the relationship between students' learning satisfaction and self-efficacy during the emergency transition to remote learning amid the coronavirus pandemic: A cross-sectional study

**DOI:** 10.1007/s10639-021-10644-7

**Published:** 2021-07-13

**Authors:** Monira I. Aldhahi, Abdulfattah S. Alqahtani, Baian A. Baattaiah, Huda I. Al-Mohammed

**Affiliations:** 1 Department of Rehabilitation Sciences, College of Health and Rehabilitation Sciences, Princess Nourah Bint Abdulrahman University, P.O. Box 84428, Riyadh, Kingdom of Saudi Arabia 11671; 2 Department of Rehabilitation Sciences, College of Applied Medical Sciences, King Saud University, P.O. Box 10219, Riyadh, Kingdom of Saudi Arabia 11433; 3 Department of Physical Therapy, Faculty of Medical Rehabilitation Sciences, King Abdulaziz University, P.O. Box 80200, Jeddah, Kingdom of Saudi Arabia 21589; 4 Department of Radiological Sciences, College of Health and Rehabilitation Sciences, Princess Nourah Bint Abdulrahman University, P.O. Box 84428, Riyadh, Kingdom of Saudi Arabia 11671

**Keywords:** Remote learning, Education, Self-efficacy, Technology, Students’ satisfaction, COVID-19 pandemic

## Abstract

The overarching objective of this study was to assess learning satisfaction among students and to determine whether online-learning self-efficacy was associated with online learning satisfaction during the emergency transition to remote learning. This cross-sectional study involved a survey distributed to 22 Saudi Arabian universities. The survey used in this study consisted of an online learning self-efficacy (OLSE) questionnaire and an electronic learning (e-learning) satisfaction questionnaire. A total of 1,226 respondents voluntarily participated in and completed the survey. Students in medical fields made up 289 (23.6%). A Kruskal–Wallis H test and a chi-square test were used to compare the student’s satisfaction based on the educational variables. Spearman’s correlation and multiple linear regression analyses were performed to assess the association between self-efficacy and satisfaction. The findings revealed degrees of satisfaction ranging between high satisfaction and dissatisfaction. The majority of students (51%) expressed high satisfaction, and 599 students (49%) reported experiencing a low level of satisfaction with e-learning. A comparison of groups with low and high satisfaction scores revealed a significant difference in the OLSE. High satisfaction was positively correlated with the OLSE domains: time management, technology, and learning. The OLSE regression analysis model significantly predicted satisfaction. It showed that the model, corrected for education level and grade point average of the students, significantly predicted e-learning satisfaction (F = 8.04, R^2^ = 0.59, *p* = .004). The study concluded that students’ satisfaction with the e-learning experience is influenced by e-learning self-efficacy. The study’s findings lead to the practical implications and identify the need to improve the remote learning, time management and technology self-efficacy to enhance students’ satisfaction.

## Introduction

The outbreak of the novel 2019 coronavirus disease (COVID-19) led to the rise of emergency remote learning as a prudent attempt to contain the spread of the virus. Emergency remote learning is a conceptual model of learning that comprises a quick transformation from face-to-face courses to online delivery, providing improvised solutions to accommodate unexpected circumstances (Hodges et al. 2020; Morgan 2020). Online education requires careful instructional design and the contemplation of different policies (Branch and Dousay 2015). However, the careful design process may be absent or partially implemented in most cases during this emergency shift (Branch and Dousay 2015). Emergency remote learning requires support, not just instructionally, but with co-curricular involvement and other system supports. The infrastructure around remote education should be sufficient to support the student’s success. In most cases, the careful design process may be absent during the sudden shift due to the unprecedented outbreak of COVID-19.

Electronic learning (e-learning) in the Kingdom of Saudi Arabia (KSA) has been facilitated by online learning technology that has allowed 100% of universities to continue education remotely (Al-Asmari and Khan 2014). Most universities invested in a large team of specialists to enable the remote learning experience and provide a unique preparation program for students and faculty. An array of technological tools that enhance learning interactions through learning management systems, such as video conferencing, discussion forums, threads, or prerecorded videos, have been utilized to deliver distance education (Al Ghamdi 2017; Alsaysi 2016). However, it is unclear to what extent students’ remote learning satisfaction and self-efficacy in Saudi Arabian universities are affected by this emergency shift.

Student satisfaction has been defined as “a short-term attitude resulting from an evaluation of students’ educational experience, services, and facilities.” (Weerasinghe and Fernando 2017, p 533). Student satisfaction might be negatively affected by taking online courses as compared to traditional ones. Online learning has been reported to have a negative impact on students’ satisfaction (Cole et al. 2014) and performance (Xu and Jaggars 2013). A previous study investigating student satisfaction indicated a significant positive correlation between students’ learning satisfaction and academic performance (Ko and Chung 2014). The degree to which students were satisfied with e-learning has been acknowledged to mediate students’ learning experiences (Atchley et al. 2013). Learning satisfaction is a key indicator of students’ learning performance (Alavi and Vogel 1997; Maki et al. 2000); moreover, gauging learning satisfaction is essential in understanding students’ perspectives of their learning experiences (Erez and Judge 2001; Sockalingam 2013). In contrast, many academic leaders found that the outcomes in online learning were similar to or better than in-person classes (Allen and Seaman 2013; Atchley et al. 2013). The complete transition to virtual classes due to COVID-19 restrictions has revived the need to explore the factors that might moderate student’s e-learning satisfaction.

A meta-analysis comparing student satisfaction with distance education to traditional classrooms revealed different factors contributing to student learning satisfaction. Regarding to distance education, the digital literacy levels, the learner’s engagements, the instructor support and guidance and the course design have been mentioned to positively correlated with learner’s satisfaction (Allen et al. 2002). Instructor support has been shown to play an essential role in affecting student satisfaction in the online environment (Bolliger 2004). Students’ interaction has also been also mentioned as a contributing factor to learners’ satisfaction (Moore 1989). In general, personal factors such as student personality, attitudes toward the technology, prior use experience, and skill have been noted to influence learners’ satisfaction (Bolliger 2004). Autonomy and students perception of their ability to carry out task control their own learning have been essential keys to affecting student satisfaction (Seiver and Troja 2014).

Apart from online learning satisfaction is the online learning self-efficacy (OLSE) which required further elucidation. Self-efficacy is commonly researched construct in traditional and online educational environments. Self-efficacy is conceptualized as an individual’s belief in the capabilities and skills required to produce the desired outcomes adequately (Bandura 1986a). It is crucial in academic learning, as it determines students’ performance and outweighs other cognitive processes (Geitz et al. 2016; Hodges 2008; Schunk 1991). Bandura’s social cognitive theory recognizes academic self-efficacy as a force of the learning system that influences an individual’s development (Bandura 1986b; Zimmerman et al. 1992). Academic self-efficacy may moderate students’ e-learning experiences and significantly impact their conceptual thinking and e-learning satisfaction. However, when academic-related self-efficacy is jeopardized, students are more likely to disengage from an assigned task and cease trying (Lee et al. 2020), which may influence the learning satisfaction.

Considering academia-related self-efficacy, Shen et al. (2013) investigated the OLSE and its related variables among students. These variables include technology and learning self-efficacy. The authors found that students’ sex, academic level, and the number of virtual courses in which they were enrolled accounted for only 7% of the variation in students’ OLSE to complete the course. They also found that students’ academic level was a significant predictor of OLSE related to the handling of digital technological tools. The author explored the relationship between self-efficacy and student satisfaction and found that OLSE predicted students’ online learning satisfaction (Shen et al. 2013). In a study investigating the relationship between learner satisfaction and self-efficacy of 440 participants, the author revealed that the self-efficacy is a significant predictor of students' learning satisfaction in online learning environments (Womble 2008). Likewise, previous study attempted to understand learner satisfaction in the context of online learning as well (Lin et al., 2008). For students enrolled in a distance learning program, the authors found that self-efficacy significantly impacted online learning satisfaction (Lin et al. 2008). Moreover, the computer self-efficacy plays a significant role in determining student learning satisfaction; it also predicts students’ intent to take future web-based courses (Lim 2001).

However, with the abrupt shift to online learning due to the COVID-19, learning practices and learners’ experience, particularly among Saudi Arabian universities, might be disrupted. It is also unclear to what extent university students’ OLSE influences their e-learning satisfaction. The literature is lacking, and little is known about the relationship between students’ OLSE and their e-learning satisfaction in KSA university students during COVID-19. It is unknown whether students’ OLSE plays a role in facilitating the educational transformation in KSA universities. Therefore, the intended research question to accomplish the objective of the study is: Does online learning self-efficacy contribute to e-learning satisfaction of among university students? To answer this question, our specific aims were:

1. Assessing the extent of students’ e-learning satisfaction.
2. Investigating the dimensions of online learning self-efficacy among college students at Saudi Arabian universities.
3. Understanding the predicating effect of online learning self-efficacy on e-learning satisfaction.

We hypothesized that the domains of learning self-efficacy would positively influence OLSE satisfaction among college students in Saudi Arabian Universities. Exploring the relationship between learning self-efficacy and OLSE satisfaction was important as e-learning satisfaction might affect the students’ academic performance.

## Materials and methods

### Study design and participants

A cross-sectional study was conducted in May 2020 during the COVID-19 lockdown in the KSA. After a structured survey development process, the survey was distributed to undergraduate and postgraduate students in Saudi Arabian universities using an online survey (SurveyMonkey®; Palo Alto, CA, USA). The target population included college students in either sophomores, junior, or senior year. Students were older than 18 and studying full-time in various majors, including medical and non-medical fields. The students were excluded if they were taking traditional classes or blended *learning*. A convenience sample consisting of 1,226 students at 22 universities in KSA responded to e-mails. At the beginning of the survey, the study protocol, procedures, and participants’ rights were explained. Participants’ demographic characteristics are presented in Table 1.

**Table 1:** Demographic characteristics of study respondents

Variables	N	%
Gender		
Male	215	27.5
Female	1011	82.4
Field of the study		
Medical	289	23.6
Non-medical	937	76.4
Age categories		
18–25	1023	83.4
26–35	164	13.4
> 35	39	3.14
Educational level		
Diploma	45	3.7
Bachelor’s	1019	83.1
Postgraduate	162	13.2
Grade point average (GPA)		
90–100	657	53.5
80–89	379	30.6
70–79	166	13.5
< 70	27	2.14

Data are presented as frequency (N) and percentage (%)

### Ethical considerations

Before data collection began, this study was registered and approved by an appropriate institutional review board [blinded for review] (no. 20–017). In the online survey, all respondents were asked to provide informed consent before completing the survey. The information and responses were treated as confidential and anonymous.

### Study instrumentation

The online survey was sent to students via e-mail with a link to the study description. The respondents were then asked to forward the e-mail to their acquaintances. A reminder to participate in the survey was sent biweekly to maximize the response rate during the data collection period. Once respondents provided consent, they were given access to the online survey. The survey took approximately 10 min to complete. Data were collected between May and June 2020.

The survey consisted of a request for demographic information, an OLSE questionnaire, and an e-learning satisfaction questionnaire. The survey’s design was adapted from studies by Zimmerman and Kulikowich (2016) on self-efficacy and by Wang (2003) on e-learning satisfaction, which have been extensively used in related research.

The OLSE questionnaire developed by Zimmerman and Kulikowich (2016) is a 22-item instrument to assess the three constructs related to OLSE: e-learning (10 items), time management (five items), and technology (seven items) (see Appendix). (Zimmerman and Kulikowich 2016). The scale items were ranked using a six-point Likert scale, where 1 = strongly disagree and 6 = strongly agree. For each construct, items responses on a six-point Likert scale were averaged to obtain a composite response. The overall OLSE was integrated into a single composite response by taking the average for each response of the 22 items that displayed on the Likert scale. The reliability results of the scale revealed Cronbach’s alphas of 0.89 for the 10-item online learning environment subscale, 0.85 for the five-item time management subscale, and 0.84 for the seven-item technology use subscale.

The e-learning satisfaction questionnaire was adapted from Wang (2003). It had a Cronbach’s alpha of 0.95, indicating excellent reliability. The satisfaction measurement had 20 items, ranked on a five-point Likert scale from 1 (“strongly disagree”) to 5 (“strongly agree”). This item in this scale is a series of Likert-type that when combined give a composite score that describes the level of satisfaction. A higher score indicates a higher level of satisfaction. A pilot study was conducted to examine the reliability of the questionnaire, which was tested with a group of 50 participants using Cronbach’s alpha. The results yielded excellent reliability (Alpha = 0.97).

### Statistical analysis

A Shapiro–Wilk test was conducted to test for the normality assumption of the data. Data were presented as the median and interquartile range (IQR) for continuous variables and frequency and percentage (%) for categorical variables. The normality assumption was violated, and none of the major outcomes followed a normal distribution; therefore, baseline data were analyzed. For categorical demographic characteristics, the Pearson chi-square test was conducted. In addition, the differences in continuous variables in the satisfaction scale for the educational characteristics (GPAs and education level) characteristics were assessed using the Mann–Whitney U test to compare between the two nominal categories (sex and field of study). In contrast, a Kruskal–Wallis H test was used to compare the education level and age categories. A Kruskal–Wallis H test was conducted to characterize the difference in the OLSE responses based on the Satisfaction categories (low vs. high). To test the hypothesis that OLSE is correlated with e-learning satisfaction, we used Spearman’s correlation coefficient. Multiple linear regression analyses were also conducted to identify the association between e-learning satisfaction (dependent variable) and self-reported outcomes of online self-efficacy, including learning, time management and technology self-efficacy (independent variables) after adjusting for the student’s educational level and GPA. Statistical significance was set at *p* < 0.05. The collected data were analyzed using the Stata version 16 (StataCorp, College Station, Texas 77845 USA).

## Results

A total of 1,226 students voluntarily consented to complete the survey, and 30 respondents declined to participate. The results showed that the median student satisfaction with e-learning was 69, and the IQR was 21. Of the 1,226 respondents, 599 (49%) reported a low level of satisfaction that was below the median value, and 627 (51%) expressed high satisfaction with e-learning, as reflected by a value above the median. There was no significant difference in the median e-learning satisfaction between the medical and non-medical fields (68 [IQR = 21] and 69 [IQR = 21], respectively; *p* = .7). Table 2 shows no differences in e-learning satisfaction between the education level and the GPAs categories (*p* = .5).

**Table 2:** Comparing students’ electronic learning satisfaction

Variable	Electronic-learning satisfaction	
	Median (IQR)	*p*-value*
Educational level		
Diploma, *n* = 45	74 (30)	.59
Bachelor’s, *n* = 1019	69 (22)	
Postgraduate, *n* = 162	69 (19)	
GPA		
90—100	70 (22)	.13
80—89	68 (20)	
70—79	67 (21)	
< 70	73 (32)	

Data are presented as medians (interquartile ranges [IQR])

^*^p-value < .05 is considered significant

Students were asked to report their OLSE in three domains: learning, time management, and technology. Table 3 summarizes the descriptive analyzes of the OLSE construct with the numbers and percentages on a six-point Likert scale. It has been reported that the rate of students reporting “strongly agree to agree” on learning self-efficacy was 36%, and 27% of the student’s responses indicated that they only “slightly agree.” Furthermore, the rate of reporting “slightly disagree to strongly disagree” was 17%. In terms of self-efficacy related to time management, about 27% of the students’ responses ranged from reporting “slightly disagree to strongly disagree”. Comparing the groups with low and high satisfaction yielded a significant difference in the overall OLSE scores (*p* < .001). Nearly all the students with high satisfaction levels (98%) responded more positively regarding the OLSE than those with low satisfaction levels (61%), as shown in Table 4.

**Table 3:** Descriptive statistics concerning students’ online learning self-efficacy

Parameters^a^	Strongly agree	Agree	Slightly agree	Slightly disagree	disagree	Strongly disagree
	*N* (%)	*N* (%)	*N* (%)	*N* (%)	*N* (%)	*N* (%)
Learning	110 (9%)	339 (27%)	459 (37%)	227 (18%)	74 (6%)	20 (2%)
Time management	158 (13%)	385 (31%)	346 (28%)	246 (20%)	63 (5%)	28 (2%)
Technology	188(15%)	455(37%)	377(31%)	151 (12%)	45 (4%)	10 (1%)

Data are presented as frequency (*N*) and percentage (%)

^a^Zimmerman and Kulikowich 2016

**Table 4:** Students’ online learning self-efficacy scores based on the level of e-learning satisfaction

Variable	Overall items of OLSE response						*p*-value
	Strongly agree	Agree	Slightly agree	Slightly disagree	Disagree	Strongly disagree	
Level of satisfaction	*N* (%)	*N* (%)	*N* (%)	*N* (%)	*N* (%)	*N* (%)	
Low satisfaction (*n* = 599)	3 (.50%)	92 (15%)	268(45%)	177(29.5%)	46(8%)	13(2%)	< .001*
High satisfaction (*n* = 627)	11(18%)	304 (48%)	198(32%)	14(2%)	0	0	

Data are presented as frequency (%)

^*^p-value < .05 is considered significant

**Fig. 1:**
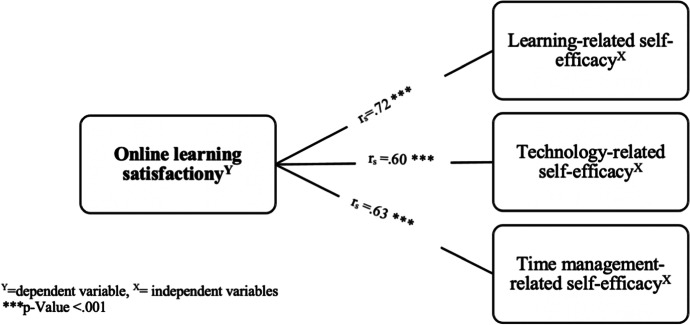
Depicts the correlation between online-learning self-efficacy of the students and e-learning satisfaction

Figure 1 depicts Spearman’s correlation coefficients (r_s_) used to assess the size and direction of the relationship between OLSE and electronic learning satisfaction. A strong positive correlation was observed between students’ e-learning satisfaction and all three OLSE domains: learning, timing, and technology. As shown in Table 5, the OLSE including, learning, technology, and time management related to self-efficacy, were a significant predictor of student satisfaction after adjusting for the student’s educational level and GPA. The model significantly predicted e-learning satisfaction (F = 8.04, R^2^ = 0.59; *p* = .004).

**Table 5:** Summary of multiple linear regression analysis of the association of online-learning self-efficacy domains on student satisfaction

Predictors^a^	$\beta$	SE	95% CI	p*-*value
Learning	8.93	.58	[7.79, 10.07]	< .001*
Time management	2.15	.42	[1.31, 2.97]	< .001*
Technology	3.22	.54	[2.16, 4.27]	< .001*

^*^p-value < .05 is considered significant. CI = confidence interval. SE = standard error. β = beta coefficients

^a^Control variables include GPAs and education level

## Discussion

This study aimed to explore students’ satisfaction with e-learning and its relationship to all aspects of learning self-efficacy, time management, and technology during the sudden shift to remote learning caused by the COVID-19 pandemic. The preliminary findings revealed promising results that students’ satisfaction with the e-learning experienced at KSA universities tended to be influenced by OLSE. Students, on average, experienced moderate satisfaction with their remote learning experience through the learning management system. Most students were satisfied, pointing to a correlation between OLSE domains and online e-learning satisfaction. This further suggests that a strong sense of OLSE enhances students’ e-learning satisfaction with the online learning environment. These findings provide important clues that might promote student satisfaction and develop a better e-learning experience by embracing students’ self-efficacy.

Student satisfaction is an indispensable aspect of learning, as it is related to academic performance and continued participation in online classes. Students are now more accepting of online learning opportunities than they were in previous years (Alahmari 2017). This study revealed moderate levels of satisfaction with online learning experiences. Student satisfaction might be influenced by course quality which is a critical mediator that strongly influences student satisfaction (Alqurashi 2019; Browne 1998). However, examining this aspect was beyond the scope of the present study.

Several factors may have influenced student satisfaction, which could explain our findings. First, the design of courses and pedagogical clarity may affect students’ satisfaction and retention rates. Researchers concur that the quality of electronic-based educational courses shapes students’ learning experiences and determines whether favorable learning opportunities are provided (Alqurashi 2019; Janicki and Liegle 2001). Second, communication and interaction through the medium, not only between educators and students but also between classmates, during online courses significantly impacts student satisfaction (Swan 2001).

Related literature shows that the effect of interaction and communication on student satisfaction is explained by the transactional distance theory (TDT) (Bolliger and Halupa 2018; Gavrilisr et al. 2020; Weidlich and Bastiaens 2018). TDT is the cognitive gap between the teacher and learners; it functions as the interplay of the structural method and the autonomy of the learner, facilitating dialog that underpins the complex practice of learning processes at a distance (Keegan 2005; Moore 1997). Educational researchers have revealed that teachers’ immediacy, whether in the form of teachers’ verbal or non-verbal (facial expression, eye contact) communication, can lessen the psychological distance between the communicators and lead to a great learning experience (Moore, 1997; Keegan, 2005). Satisfaction with the virtual environment, which necessitates high-order thinking, reflects students’ learning self-efficacy and sense of transactional distance (Zilka et al. 2019). Shortening the transactional distance by facilitating dialog will boost the learning experience and help enrich learners’ online self-efficacy (Delgaty 2018). Furthermore, interactions with students in contemporary online learning environments are a function of multidimensional constructs known as the community of inquiry (CoI) model. This reflects the interplay of cognitive presence, social presence, and teaching and cognitive presence (Castellanos-Reyes 2020). A study noted that the CoI framework is a predictor of learner satisfaction (Joo et al. 2011). Therefore, it is critical to facilitate communication and interaction during e-learning between the instructor and students, and between students to improve student satisfaction.

The findings of this study shed light on the significant association of student OLSE with student satisfaction. The OLSE of students contributes to learning, mental health, and motivation (Nie et al. 2011). The concept of self-efficacy has been recognized in several fields. Self-efficacy related to learning, such as e-learning and handling electronic technology and time management, may be influenced by educational components. Previous studies indicate that students with strong academic self-efficacy experience less academic anxiety and stress (Nie et al. 2011) and achieve academic tasks more successfully (Elias and MacDonald 2007; Gore 2006; Hejazi 2009). Gunawardena et al. (2010) found that the strongest predictor of student satisfaction was online self-efficacy, consistent with this study’s findings. Our study demonstrated that students’ OLSE is a strong predictor of student satisfaction, consistent with a previous study that showed higher OLSE to be an essential factor for higher satisfaction with e-learning (Lee and Hwang 2007).

The level of satisfaction and OLSE can be explained by the universities’ support system. Most universities in the KSA invest in an ecosystem of learners that promotes remote education (Al-Asmari and Khan 2014). Despite the transition in a narrow preparation window, university support personnel are available to help faculty members and students implement online learning. Universities and the Ministry of Education have implemented the instructional support unit to help students and faculties partner with faculty experts to endorse digital fluency and help students and faculty develop skills to manage the online environment. However, there is a possibility of suboptimal implementation of online learning due to the inevitable transition to emergency remote learning, surpassing education system capacities and influencing our findings.

Regarding the self-efficacy of using the technology use, in the present study, approximately 17% of the students expressed less self-efficacy related to using the technology use in learning, which implying a lack of skills required to deal with e-learning systems. These students could be at risk of stress and depression because they may face difficulties completing their e-learning tasks, requiring intensive training to learn how to utilize e-learning systems (Alqurashi 2016; Martin et al. 2010). Future studies might investigate the relationship between students’ e-learning skills, stress and depression levels, as these factors were beyond the scope of this study.

Various factors may play essential roles in shaping students’ OLSE, such as the instructor’s behavior. Previous research has clearly stated that there is a direct relationship between the behavior of educators in the classroom and students’ self-efficacy, as negative behavior directly affects students’ self-efficacy (Kim et al. 2018; Mitchell and DellaMattera 2010). Learners in education experience through synchronous computer-mediated communication the need for both autonomy and teacher presence. Teacher presence is one of the elements of the CoI framework of contemporary online learning and teaching, which influences student satisfaction (Akyol 2012; Garrison and Arbaugh 2007; Garrison et al. 1999). The CoI framework guides instructional methods, which may profoundly influence online learning satisfaction and continued education. These findings suggest that the positive behaviors of teachers toward students should be encouraged as they affect students positively in molding their OLSE.

Another point to considered is the students’ academic performance. There is a possibility that the students’ academic performance as defined by GPAs may influence the students’ OLSE Our previous study investigating the predicator of OLSE found a positive association between students’ GPA and time management related to self-efficacy (Aldhahi et al. 2021). The students with excellent GPAs (defined as percentage grade ≥ 90%) demonstrated good time management self-efficacy than their counterparts with good and fair GPAs. A growing number of studies, It was found that higher self-efficacy increased the students mental effort related to academic learning and performance (Chemers et al 2001; Margolis and McCabe 2003). In contrast, it has been reported that lower levels of academic self-efficacy among students explain the decline in their academic performance and lack of the commitment to achieve the academic duties (Bandura 1997).

The current study identified significant positive relationships between the domains of OLSE and students’ e-learning satisfaction. Our results are consistent with those of Jan (2015), who showed a significant positive relationship between the overall domains of academic self-efficacy and satisfaction among 103 students at a university in the United States. However, the relationship the association disappeared when the analysis compared only the technology domain and satisfaction (Jan 2015). This difference could be attributed to the sample size and the differences in the methods used to assess the outcomes and the operational definition of self-efficacy. It was not related to OLSE. Another study found a significant relationship between technology self-efficacy and satisfaction among employees who took an online training course (Womble 2006). This implies that students with technological skills are more satisfied with e-learning environments than those without e-learning. However, a comparison is inappropriate because our study population was comprised of students enrolled at universities.

## Limitations and future research

Although this study demonstrated an understanding of the extent of OLSE predication to students’ satisfaction in electronic-based learning environments, several limitations should be noted. First, according to Wang (2003) and Zimmerman and Kulikowich (2016), most of the measures used by researchers to assess satisfaction and student perceptions of self-efficacy are self-reported measures. Self-reported questionnaires may propagate reporting bias, as students might not judge their skill levels accurately. Therefore, future research should consider utilizing integrated methods, such as combining quantitative and qualitative designs, to obtain complete information and minimize such bias. Thus, we used an online learning scale that measures well-planned online course self-efficacy. However, our study focused on emergency remote teaching, which should be considered when interpreting our findings.

There was a variation between universities in the planning and preparation of online learning. Some universities have activated e-learning even before the pandemic, which may affect our findings. Nevertheless, we did not investigate how familiar universities were with e-learning. Third, other domains of self-efficacy, such as metacognition (Moores et al., 2006), could explain student satisfaction and should be introduced in the model as a mediator in future research. Fourth, the sample in this study included only willing respondents from the KSA at public universities, which may limit the generalizability of the results. Including students from different populations would help to confirm and refine the mediating effect of OLSE on student satisfaction. The quality of education and curriculum, interaction and communication between classmates and instructors, internet connection, and high-quality devices could be important factors affecting student satisfaction. These factors could be the reasons behind the relatively high percentage of ‘low satisfaction.’ However, we could not confirm this because we did not control for them as a covariate. Therefore, controlling for these factors in future studies should be encouraged.

Moreover, COVID-19 led to emergency remote learning, which should not be equated with online learning. Accordingly, the findings might not apply to other learning settings, such as hybrid or blended courses. Future research should consider the variations between learning settings, comparing synchronous and asynchronous learning formats of learning, or traditional and hybrid learning. Such future research could help educators and decision-makers to enhance the e-learning environment, which is likely to be utilized more frequently in the future. With proper planning, universities need to assess strengths and weaknesses to best prepare for future online learning needs.

## Conclusions

This study revealed that the three domains of OLSE—learning, time management, and technology—played salient roles in student satisfaction during the emergency shift to remote learning owing to the COVID-19 pandemic. Students’ judgment of their ability to complete online courses is critical for providing a successful remote learning-based approach. These empirical findings add to the fundamental knowledge of both educators and decision-makers as a method of assessing academic self-efficacy in online learning environments. Our results may also help instructors and the education sector provide proactive strategies and approaches to improve all domains of students’ self-efficacy to embrace the dynamic remote learning-based approach. Institutions should take this opportunity to evaluate how well they implemented remote learning to maintain the continuity of education, especially under any crisis condition.

## Data Availability

The datasets generated during and/or analyzed during the current study are available from the corresponding author on reasonable request.

## Appendix

**Table:**

Online Learning Self-Efficacy (OLSE)^a^	Learning	Time	Technology
1. Navigate online course materials efficiently			Item1
2. Find the course syllabus online			Item 2
3. Communicate effectively with my instructor via e-mail			Item 3
4. Communicate effectively with technical support via email, telephone, or live online chat	Item 1		
5. Submit assignments to an online drop box			Item 4
6. Overcome technical difficulties on my own	Item 2		
7. Navigate the online grade book			Item 5
8. Manage time effectively		Item 1	
9. Complete all assignments on time		Item 2	
10. Learn to use a new type of technology efficiently	Item 3		
11. Learn without being in the same room as the instructor	Item 4		
12. Learn without being in the same room as other students	Item 5		
13. Search the Internet to find the answer to a course-related question			Item 6
14. Search the online course materials			Item 7
15. Communicate using asynchronous technologies (discussion boards, e-mail, etc.)	Item 6		
16. Meet deadlines with very few reminders		Item 3	
17. Complete a group project entirely online	Item 7		
18. Use synchronous technology to communicate with others (such as Skype)	Item 8		
19. Focus on schoolwork when faced with distractions		Item 4	
20. Develop and follow a plan for completing all required work on time		Item 5	
21. Use the library’s online resources efficiently	Item 9		
22. When a problem arises, promptly ask questions in the appropriate forum (e-mail, discussion board, etc.)	Item 10		

^a^ Zimmerman, W. A. and Kulikowich, J. M. (2016). Online Learning Self-Efficacy in Students with and Without Online Learning Experience. *American Journal of Distance Education*, *30*(3), 180–191. 10.1080/08923647.2016.1193801
